# The late response of rat subependymal zone stem and progenitor cells to stroke is restricted to directly affected areas of their niche^☆^

**DOI:** 10.1016/j.expneurol.2013.06.025

**Published:** 2013-10

**Authors:** Ilias Kazanis, Natalia Gorenkova, Jing-Wei Zhao, Robin J.M. Franklin, Michel Modo, Charles ffrench-Constant

**Affiliations:** a MRC Cambridge Centre for Stem Cell Biology and Regenerative Medicine and Department of Veterinary Medicine, University of Cambridge, Cambridge, UK; b Department of Neuroscience, Institute of Psychiatry, King's College London, London, UK; c MRC Centre for Regenerative Medicine, Centre for Multiple Sclerosis Research, University of Edinburgh, Edinburgh UK

**Keywords:** Neurogenesis, Neural stem cells, Progenitors, Subependymal zone/subventricular zone, Stroke, Ischaemia, Proliferation

## Abstract

Ischaemia leads to increased proliferation of progenitors in the subependymal zone (SEZ) neurogenic niche of the adult brain and to generation and migration of newborn neurons. Here we investigated the spatiotemporal characteristics of the mitotic activity of adult neural stem and progenitor cells in the SEZ during the sub-acute and chronic post-ischaemic phases. Ischaemia was induced by performing a 1 h unilateral middle cerebral artery occlusion (MCAO) and tissue was collected 4/5 weeks and 1 year after the insult. Neural stem cells (NSCs) responded differently from their downstream progenitors to MCAO, with NSCs being activated only transiently whilst progenitors remain activated even at 1 year post-injury. Importantly, mitotic activation was observed only in the affected areas of the niche and specifically in the dorsal half of the SEZ. Analysis of the topography of mitoses, in relation to the anatomy of the lesion and to the position of ependymal cells and blood vessels, suggested an interplay between lesion-derived recruiting signals and the local signals that normally control proliferation in the chronic post-ischaemic phase.

## Highlights

• Neural stem cells respond transiently to ischaemia.
• Progenitors respond chronically to ischaemia.
• Neural stem/progenitor cells of the subependymal zone respond only in areas of damage to the niche.
• Ischaemia affects the cytoarchitecture of mitoses in the ageing niche.
• Density of microglia is decreased in the ageing niche.

## Introduction

The largest neurogenic area of the adult rodent and human brain is the subependymal zone (SEZ), located at the lateral wall of the lateral ventricles, in which relatively quiescent neural stem cells (NSCs) generate actively dividing progeny (Lois and Alvarez-Buylla, 1994). In rodents, SEZ-born neuronal progenitors have the capacity to migrate long distances, through a specialized route called rostral migratory stream (RMS), in order to reach their final destination within the olfactory bulb (OB) (Riquelme et al., 2008). Experimental studies have shown that neurons and glia are also born at the SEZ in response to focal ischaemic lesions that model stroke in humans (Li et al., 2010a; Zhang et al., 2001, 2004) with many of these newly-generated cells migrating towards the infarcted areas (Hou et al., 2008; Jin et al., 2010; Thored et al., 2006, 2007; Yamashita et al., 2006). Proliferation in the SEZ peaks at around 1 week post-ischaemia, though SEZ-driven striatal neurogenesis persists for at least 4 months and is thought to correlate with spontaneous recovery during this sub-acute phase (Thored et al., 2006). Although only limited evidence exists demonstrating the potential of SEZ-derived newborn cells to develop into viable and functional neurons (Hou et al., 2008; Li et al., 2010a; Thored et al., 2006), the experimental ablation of endogenous neurogenesis in a transgenic mouse in which progenitors of neuronal commitment were depleted, compromised early post-ischaemic neuroprotection (Jin et al., 2010; Sun et al., 2012; Wang et al., 2012). Conversely, exogenous stimulation of neurogenesis through increased Wnt-3A expression or administration of retinoic acid enhanced tissue protection (Plane et al., 2008; Shruster et al., 2012). These results indicate that neurogenesis from the SEZ stem cell niche may be important for enhanced tissue preservation after stroke by the generation of cells with neuroprotective properties, and that it therefore constitutes a valid target for therapeutic interventions. However, in order to fully appraise its potential to be used in post-ischaemia recovery strategies, further analysis of its response after such insults is required. This includes investigating: a) the identity of the cell populations that respond (stem cells and/or their progeny), as has been done in other adult stem cell systems (Mascre et al., 2012; Simons and Clevers, 2011), b) the level of response (time frame, cell numbers) and c) the anatomy of the response (e.g. the fraction of the niche that becomes activated). In this study we map and quantify the activation of the SEZ during sub-acute and late post-ischaemic stages (4–5 weeks and 1 year, respectively), which are under-investigated though medically relevant in terms of recovery (Markus et al., 2005). We calculate the fraction of the niche responding to focal ischaemia and explore separately the mitotic activation of stem and progenitor cells. Finally, we assess the effects of ischaemia on the structure of the specialized microenvironment of the niche, focusing on the positioning of dividing progenitors in relation to two major structural elements of the SEZ: blood vessels and the ependymal cell layer/cerebrospinal fluid interface; as well as on the response of macrophages of the innate and blood-born immune system.

## Materials and methods

### Animals, experimental stroke and AraC treatment

Adult male Sprague–Dawley rats were used and all experiments were performed in accordance with the UK Animals (Scientific Procedures) Act 1986. In order to model focal ischaemia stroke in humans, ischaemia was induced by middle cerebral artery occlusion (MCAO) for 1 h, as previously reported (Modo et al., 2002). Briefly, animals were anaesthetised with isofluorane and temporary ligatures were placed on the ipsilateral external and common carotid to stop the flow of blood to the internal carotid artery. The tip of the thread (Doccol) was advanced 18–20 mm from the cervical carotid bifurcation or until reaching resistance from the ostium of the middle cerebral artery in the circle of Willis. Occluded animals were re-anaesthetised in order for the thread to be removed. For the sub-acute post-surgery stage, 260–280 g adult rats (2–3 months old) were operated (sham and ischaemia groups) and were killed 4 or 5 weeks later. Because all analyses performed in tissue derived from the two sub-acute phase time-points produced similar results (data not shown), data from 4 and 5 weeks were pooled together. For the chronic post-injury phase, another two groups of animals were operated (sham and ischaemia) and killed 1 year later. All animals were included based on the presence of a T2-hyperintense lesion using MRI according to previously published protocols (Modo et al., 2009).

AraC infusions were performed in an additional group of non-operated rats at an age equivalent to that of the sub-acute post-stroke time-point group and according to previously published protocols (Kazanis et al., 2010). AraC was infused on the surface of the brain via a cannula (BIK-II, Alzet) that was fixed on the skull (1 mm lateral and 0.5 mm rostral to bregma) and was connected to a subcutaneously implanted mini-osmotic pump (1007D, Alzet). 4% AraC (Sigma, UK) in saline, or saline alone, was infused for 7 days and animals were sacrificed 2 days after the end of the infusion. The success of the treatment was evaluated by immunostaining for the neuroblast marker Dcx, as has been previously reported in mice (Kazanis et al., 2010). In all rats included in the study AraC resulted in the complete depletion of Dcx + cells in the ipsilateral SEZ niche (as shown in Fig. S3).

### Tissue processing, imaging, data collection and statistical analysis

Animals were sacrificed by transcardial infusion of 4% paraformaldehyde (under terminal anaesthesia) and tissue was post-fixed overnight in 4% paraformaldehyde (at 4 °C). The brains were subsequently sectioned using a vibratome in 70 μm thick slices and were stored at − 20 °C in an anti-freezing solution (containing 30% glycerol, 30% Ethylene glycol in 0.5% phosphate buffered saline; reagents purchased from Sigma-Aldrich UK). Immunohistochemistry was performed using a free-floating slice protocol. Initially, the slices were treated for antigen retrieval by being incubated in a 10 mM citrate buffer (pH = 6) at 90 °C for 15 min. Slices were then incubated in blocking solution (3% BSA, 0.1% Triton X-100 in PBS) at room temperature for a minimum of 5 h. Subsequently they were incubated overnight with the primary antibodies diluted in blocking solution at room temperature, on a rocking plate. The antibodies used were the following: rabbit anti-PH3 (1/500, Millipore) and mouse anti Ki67 (1/500, Novocastra Labs, UK) to label mitotic cells, mouse anti-GFAP (1/500, Sigma) for astrocytes, rabbit anti-Dcx (1/500, Abcam, UK) for neuroblasts, rabbit anti-pan-laminin (1/100, Sigma, UK) for blood vessels and mouse anti-ED1 (CD68, 1/500, Millipore) for phagocytotic macrophages and microglia. The final step was the incubation – for a minimum of 4 h – at room temperature with the respective Alexa-conjugated secondary antibodies (Invitrogen, UK) diluted in PBS. In the case of triple stainings for GFAP, PH3 and laminin, the first two primary antibodies were incubated together and then with the respective secondary antibodies; the anti-laminin staining was performed subsequently. Biotinylated isolectin IB-4 (Invitrogen, UK) was used to stain all microglia and macrophages. Images were acquired using a Zeiss fluorescence microscope or a Leica SP5 confocal microscope and were processed using Photoshop (Adobe) software.

A range of sections was analysed from each animal, taken from the same rostro-caudal area [from bregma (= 0 mm), to 1.5 mm rostrally]. A minimum of 7 slices, of respective amongst rat anatomical areas as judged using the shape of the lateral ventricles, of the corpus callosum and of the anterior commissure, were immunostained for GFAP/PH3 and laminin. In these slices all mitotic (PH3 +) cells were identified and counted within a distance of 100 μm of the ventricular wall; this being the normal maximal width of the neurogenic niche revealed by the distribution of Ki67 + cells (Kazanis and ffrench-Constant, 2011). For each one mitotic cell, at least 3 confocal-microscopy generated optical sections were taken (using the × 63 objective and a further × 4 digital zoom) in order to assess whether it was GFAP positive or negative (see the example in Fig. S2). The boundaries of the lesion were estimated and delineated on the same slices based on the increased expression of GFAP (astrogliosis), blood vessel swelling and laminin deposition and the dorsoventral lengths of the affected areas were calculated using Image Processing and Analysis in Java software (ImageJ). For the analysis of the anatomical characteristics of the SEZ response to stroke, the neurogenic niche was divided in two halves along the dorso-ventral axis (see Fig. 5). For the analysis of PH3 + cells within the penumbra, at least three optical fields were selected in each section positioned entirely within the penumbra (as it was defined by the above-mentioned histological criteria) and at a distance higher than 500 μm from the wall of the lateral ventricle. Statistical analysis of the densities of mitotic cells for the effects of treatment (sham, ischaemia and AraC) and of the area was performed separately for NSCs and progenitors and for the two time-points, using one-way or two-way ANOVA, as required.

For the analysis of microglia/macrophages, 3 sections per animal were stained for Ki67 and IB-4, one section per animal for PH3 and IB-4 and another 3 sections per animal for ED1 and Dcx. For the analysis of the positioning of mitotic cells, measurements were performed as described previously (Kazanis and ffrench-Constant, 2011; Kazanis et al., 2010) in the same triple-stained (for PH3, GFAP and laminin to mark blood vessels) slices. For the calculation of the distance from the nearest blood vessel, an area spanning at least 30 μm in each direction around a mitotic cell was scanned with the confocal microscope, all blood vessels were identified and the shortest distance between the mitotic centre of the nucleus and the surface of the blood vessel was measured using ImageJ. Mitotic cells located near the surface of the sections (hence without a 30 μm area around them, which were less than 10% of all PH3 + cells identified) were excluded from this analysis in order to avoid missing the nearest blood vessel that might be outside the section. For the calculation of the distance from the ventricular wall, the shortest distance between the centre of the mitotic nucleus and the wall of the ventricle (the ventricular surface of the nearest ependymal cell, or the line linking the nuclei of two neighbouring ependymal cells) was calculated using ImageJ.

## Results

### Spatiotemporal profile of neural stem and precursor cell responses to focal ischaemia

**Fig. S1:**
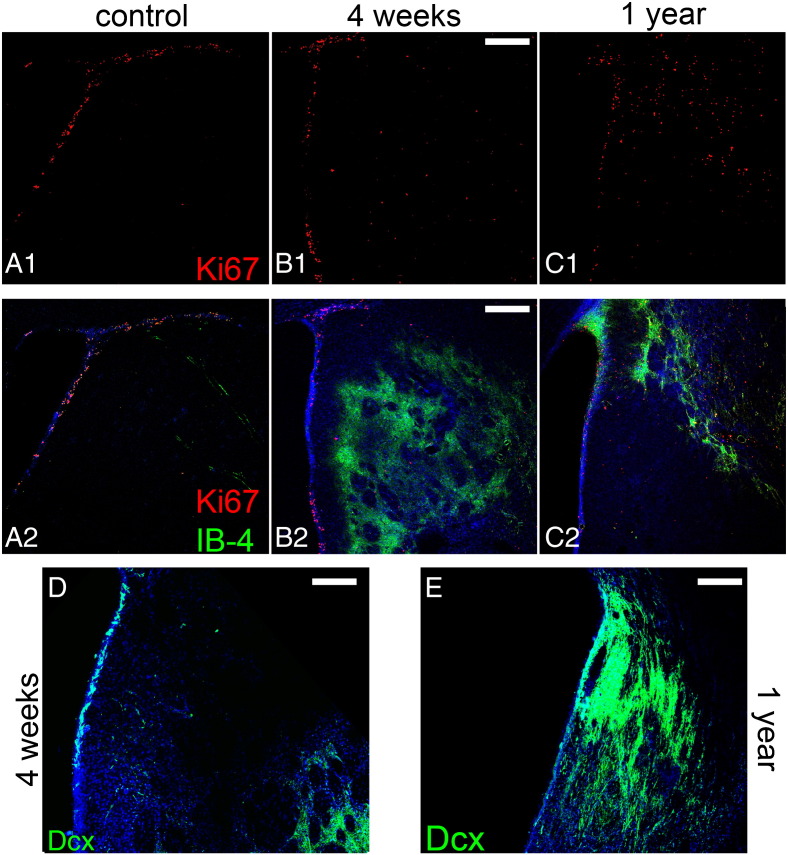
Occurrence of proliferating cells and of immature neurons at sites of lesion. Coronal sections taken from sham-operated (A), ischaemic rats at 4 weeks (B, D) and 1 year (C, E) post-injury were either double immunostained for Ki67 and isolectin B-4 (A–C), or for Dcx (D, E). (Panel A) In normal tissue all proliferating cells are located within the SEZ (approximately up to 90 μm away from the ventricular wall). (Panels B, C) After ischaemia, high numbers of Ki67 + cells appear ectopically within the striatal tissue and specifically in the damaged areas, as delineated by the increased IB-4 staining. (Panel D) At 5 weeks post-ischaemia numerous Dcx + cells have accumulated in the striatal tissue at areas proximal to the core of the lesion (at the lower right part of the image), whilst few positive cells are found at the striatal area lying between the SEZ and the lesion. (Panel E) At 1 year, high numbers of Dcx + cells are still observed at the areas of damage, either near the SEZ or deeper in the tissue. [Scale bars: 200 μm in A–C and 100 μm in D, E].

**Fig. 1:**
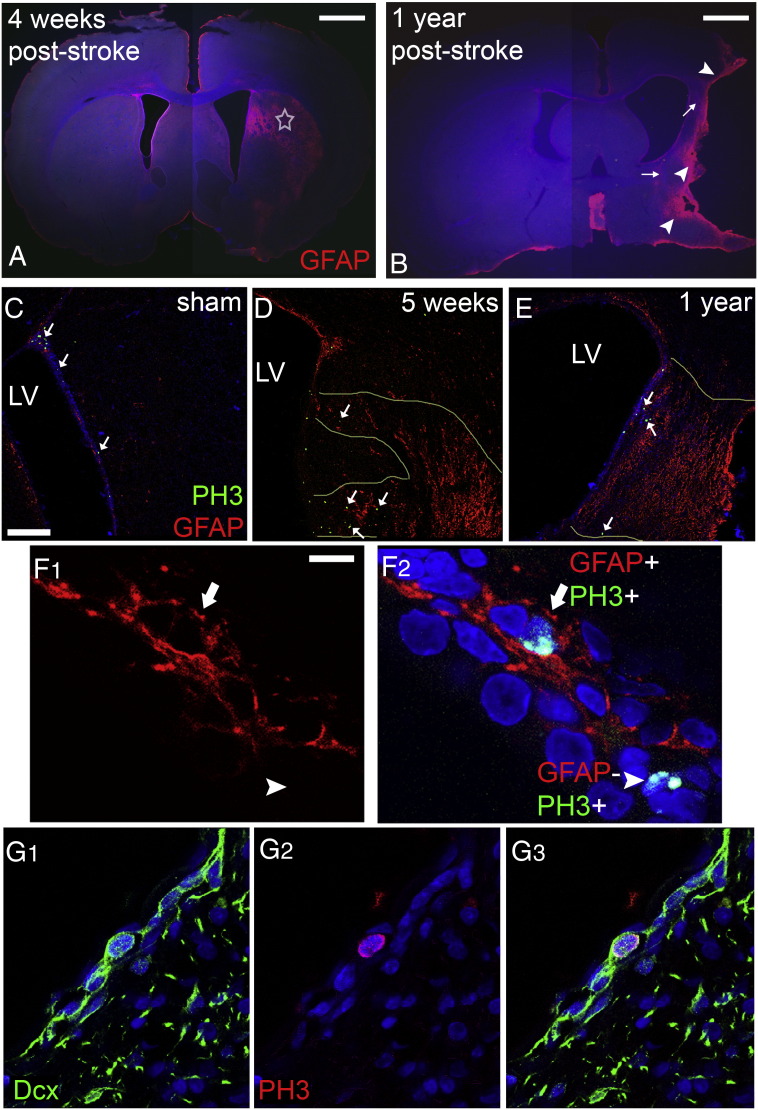
Anatomy of ischaemia-induced brain lesions. Increased GFAP immunostaining in coronal sections of rat brain 4 weeks (A) or 1 year (B) after ischaemia outlines the striatal lesion (star in A). In (B) a rare case with major striatal and cortical tissue loss is shown, with arrowheads indicating the glial scar and arrows the persistent peri-infarct astrogliosis. (Panels C–E) Microphotographs showing immunostained coronal sections, taken from sham-operated young adult (C) and post-ischaemic adult rats, either 5 weeks (D) or 1 year (E) after the insult. A gradient in the numbers of mitotic cells from dorsal (high) to ventral parts (PH3 + cells are indicated by white arrows) is observed in sham-operated animals. Ischaemia induced an increase in the numbers of PH3 + cells, especially within the areas of the tissue directly affected by injury (delineated by the pale yellow lines in D and E). (Panel F) Microphotograph showing a high magnification detail of the SEZ. Mitotic cells within the niche were immunopositive for PH3 (green in F2) and were distinguished in neural stem cells (arrow) or progenitors (arrowhead) depending on co-expression of GFAP (red). (Panel G) Microphotograph showing a high magnification detail of a mitotic cell (PH3 + in red) co-expressing Dcx (in green); thus being a neural progenitor [scale bar: 2 mm in A, B; 100 μm in C–E, 10 μm in panel F and 10 μm in G].

**Fig. 2:**
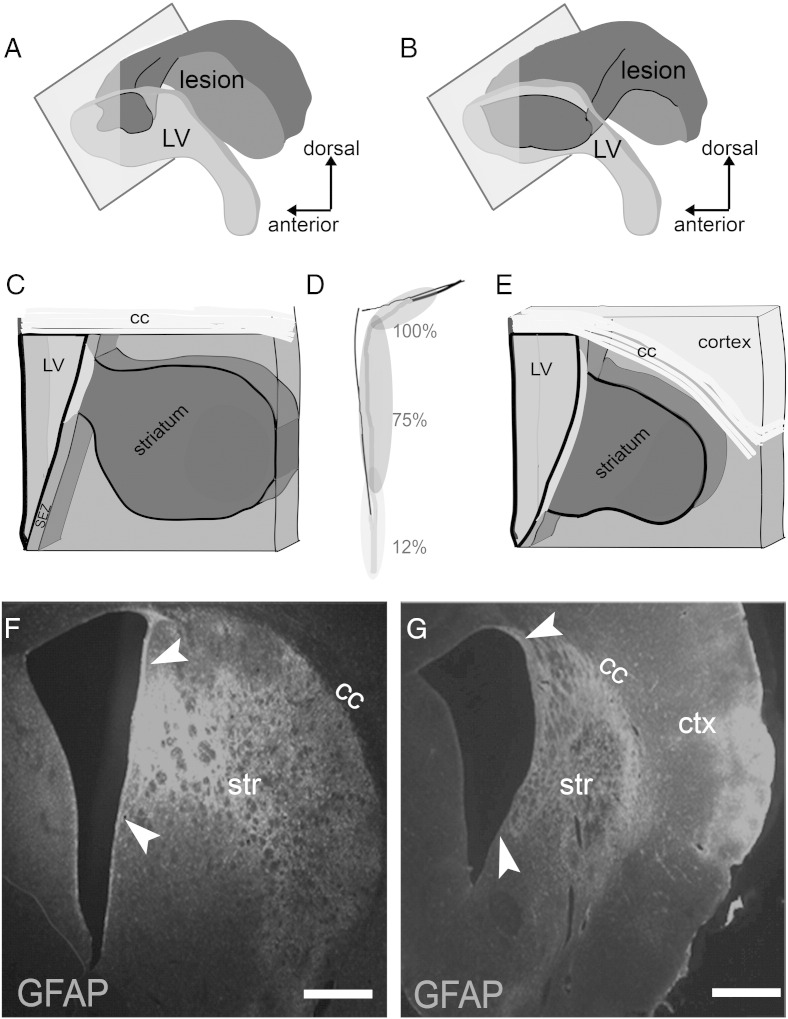
Anatomy of the lesion and of its connection with the SEZ. (Panels A–B) Illustrations summarizing results from all animals, showing the striatal lesion (dark grey) and its expansion towards the lateral walls of the lateral ventricle (the ventricle depicted in light grey). In (A) the case of a rat killed 5 weeks after ischaemia is illustrated. This was the case with the minimal observed damage to the SEZ. In (B) the case of a rat killed 1 year after ischaemia is illustrated. This was the case with the maximal observed damage to the SEZ. (Panels C and E) Illustrations of thick coronal sections showing the anatomy of the lesion in relation to the SEZ; the panels correspond to (A) and (B), respectively, in which the plane of the section is depicted as a rectangle. (D) Schematic illustration of the lateral ventricle in which the average percentage of inclusion of part or the whole of different areas of the SEZ in the ischaemic lesion is shown. Note that the dorsal part was always directly affected by ischaemia (100%), the ventral part in few cases (12%). (Panels F, G) Microphotographs of thick coronal sections of rat brains after ischaemia, in which increased GFAP immunostaining is shown to delineate the lesion (the affected part of the SEZ is highlighted by arrowheads). Note the extensive tissue loss in the striatum in (G) (the size of the striatum is less than half of that in (F)) and the expansion of the infarcted area in the cortex. The corpus callosum (cc) seems to be more resistant to degeneration. [Scale bar: 500 μm].

Surgical occlusion of the right middle cerebral artery for 1 h was performed in rats and tissue was collected for analysis in a sub-acute time-point (at 4 or 5 weeks post-insult, with data pooled together as explained in the Materials and methods section; n = 7 MCAO and n = 4 sham), as well as 1 year (n = 4 rats per group) after the insult. The area affected by ischaemia was identified based on the presence of astrogliosis (Young et al., 2012), blood vessel swelling and of the deposition of laminin, revealing significant tissue damage (and even loss) in the striatum and in some cases also in the cortex (Fig. 1). In all animals analysed the area of MCAO-induced tissue damage included parts of the lateral wall of the lateral ventricle, where the SEZ neurogenic niche is situated (Figs. 1, 2). The size of this directly affected area varied between individual cases (Fig. 2), although it more frequently included the rostral and dorsal parts of the SEZ (Figs. 2A–E). At both time-points and in all ischaemic animals, we observed the occurrence of Ki67 + (marking proliferating cells) and doublecortin + (Dcx/marking immature neurons) cells within the affected striatal tissue (Figs. S1B, C, E), in contrast to normal animals where these cells are located only in the SEZ (Figs. S1A, D). These ectopic cells were observed in various distances from the ventricular wall up to the core of the lesion.

**Fig. S2:**
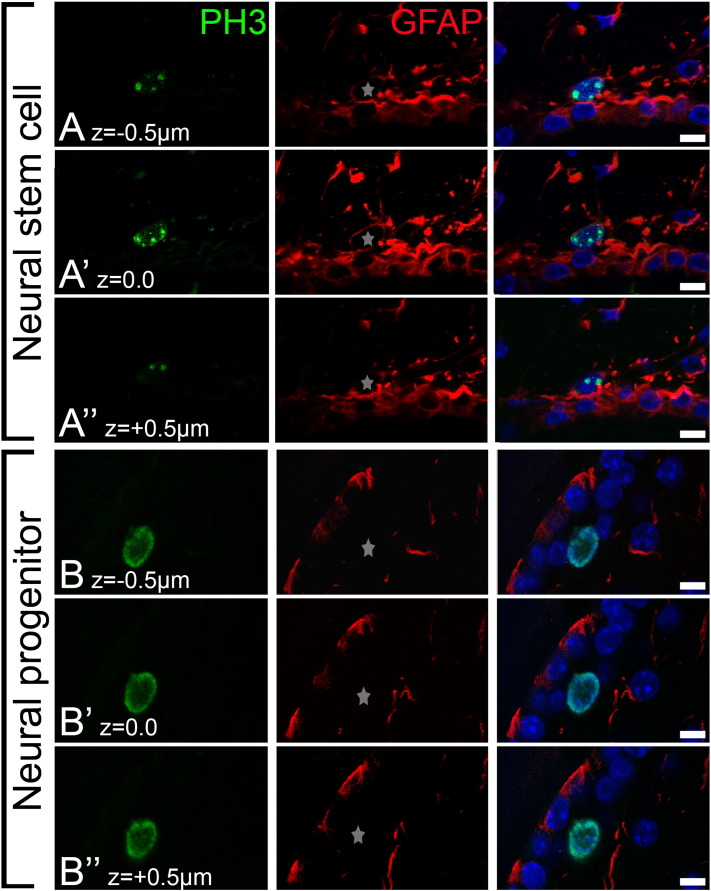
Identification of mitotic neural stem and progenitor cells. Figure illustrating the methodology that we used in order to differentially identify neural stem and progenitor cells, based on the expression of PH3 (in green) and GFAP (in red). In panels A–A″ we show one mitotic cell co-expressing GFAP, thus considered to be NSC. In panels B–B″ we show one mitotic cell that does not co-express GFAP, thus considered to be progenitor. Please note that for each PH3 + cell we used at least three optical sections (with a 0.5 μm step) in order to allocate it within the NSC or the progenitor pool; these optical sections are illustrated separately in each panel. [This is an expansion of Figs. 3B and C. Scale bar = 10 μm. The star indicates the position of the nucleus of the PH3 + cell].

**Fig. 3:**
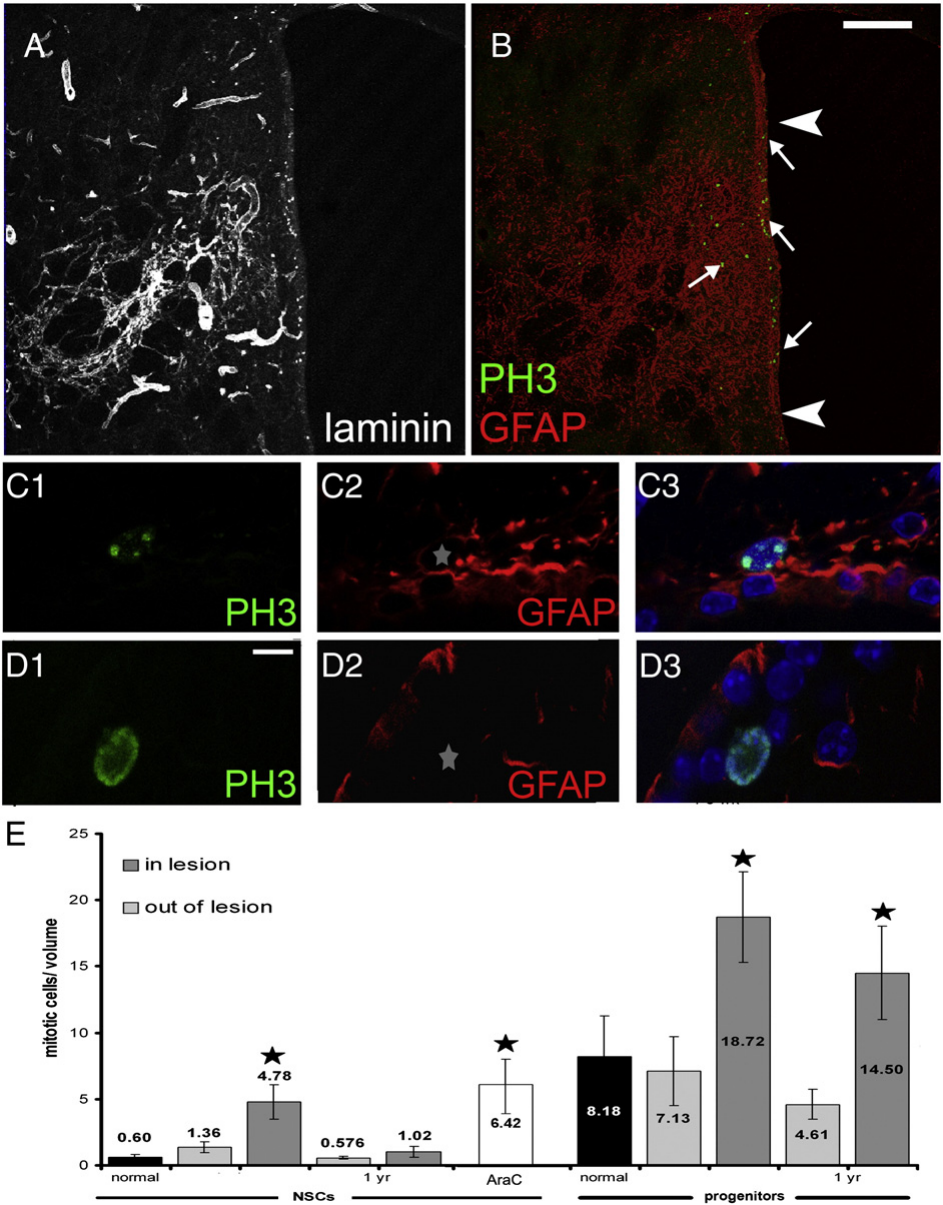
Ischaemia-induced mitotic responses. (Panels A–B) Coronal section of a rat brain, 4 weeks post-ischaemia, immunolabelled for laminin (A), or GFAP and PH3 (in B). The directly affected area of the niche is indicated by arrowheads. Note the increased occurrence of PH3 + cells specifically within the affected area. (Panel C) Example of a mitotic cell (expressing PH3 in green) that was included in the NSC pool due to the co-expression of GFAP (in red). (Panel D) Example of a mitotic cell (expressing PH3 in green) that was included in the progenitor pool due to the lack of co-expression of GFAP (in red). Please note that for each PH3 + cell we used at least three optical sections (with a 0.5 μm step) in order to allocate it within the NSC or the progenitor pool (see Fig. S2 for details on the cells shown here in panels C, D). (E) Graph showing the density of mitotic neural stem and progenitor cells in sham and ischaemic rats, separately for the SEZ parts that were directly affected by ischaemia (identified as “in lesion”) and those out of the lesion. In addition, the density of mitotic neural stem cells during regeneration of the SEZ after treatment with AraC is depicted in the white bar. Please note that in this study the relatively quiescent NSCs are identified as PH3 +/GFAP + cells and their actively dividing daughter cells (that include transit-amplifying progenitors, neuroblasts and oligodendrocyte progenitor cells and that are collectively referred to as progenitors) are identified as PH3 +/GFAP − cells. [Scale bar in A, B: 500 μm; in C, D 10 μm and the star indicates the position of the nucleus of the PH3 + cell. In (E) volume = 1 mm (length) × 70 μm (section thickness) × 200 μm (width from the ventricular wall) = 0.014 mm^3^; * = p < 0.05, compared to the respective normal value (black bars) using two-way ANOVA. Error bars depict SEM.].

The progenitor pool of the SEZ comprises relatively quiescent NSCs of astroglial morphology as well as their actively dividing daughter cells that include transit-amplifying progenitors, neuroblasts (an example is shown in Fig. 1G) and oligodendrocyte progenitor cells (all of the latter will be collectively referred to as progenitors). To investigate the mitotic response of these two separate cell populations to ischaemia we performed double-immunolabellings for Phosphohistone 3 (PH3) and GFAP. NSCs are included in the GFAP + cell population whilst neural progenitors do not express GFAP (Pastrana et al., 2009, 2011). Moreover, PH3 is expressed only during M phase of the cell cycle, in contrast to Ki67 (as used above), which also marks cells arrested in G1 or S phases. Therefore, the use of PH3 was preferred in order to distinguish the potential activated NSCs (undergoing mitosis at the time of analysis; thus being GFAP +/Ki67 +/PH3 +) (Gonzalez-Perez et al., 2009) from the “quiescent” ones (expressing Ki67, though not in M phase, since NSCs are slowly-dividing cells; thus being GFAP +/Ki67 +/PH3 −), as we have done previously (Kazanis and ffrench-Constant, 2011; Kazanis et al., 2010) (Figs. 1, 3 and S2).

**Fig. S3:**
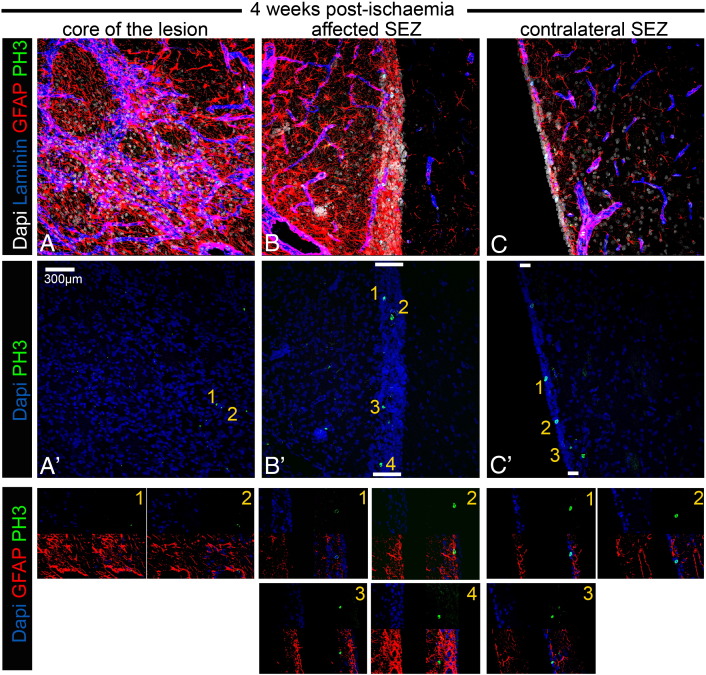
Occurrence of mitotic GFAP + cells 4–5 weeks post-ischaemia. Microphotographs showing stacks of images, generated with confocal microscopy, taken from thick coronal sections of rat brain tissue 4 weeks after MCAO. In A, high levels of immunostaining for GFAP (in red, marking astroglial cells) and for laminin (in blue, marking swelled blood vessels and deposition in the parenchyma) reveal the histological features of the ischaemia-affected area in the striatum. In B, a part of the adjacent to the lesion SEZ is depicted, characterized by the same histological features (high levels of gliosis and blood vessel swelling). In C, the contralateral (unaffected) SEZ is depicted being negative for gliosis and blood vessel swelling. The same areas are shown in the middle panel only with DNA counterstaining of nuclei (in blue) and with immunostaining for PH3 (in green). Note the rarity of mitotic figures in the gliotic area of lesion, compared to the numerous PH3 + cells observed in the affected (and thicker) SEZ. Mitotic activity is also high (but lower that in the affected SEZ) in the contralateral SEZ. In the lower panel, examples of mitotic cells included in A′, B′ and C′ are shown in higher magnification and immunostained both for PH3 (green) and GFAP (red). The numbers indicate the cell shown in more detail. The only mitotic GFAP + cell is No. 2 in B′. [White bars indicate the width of the SEZ].

**Fig. S4:**
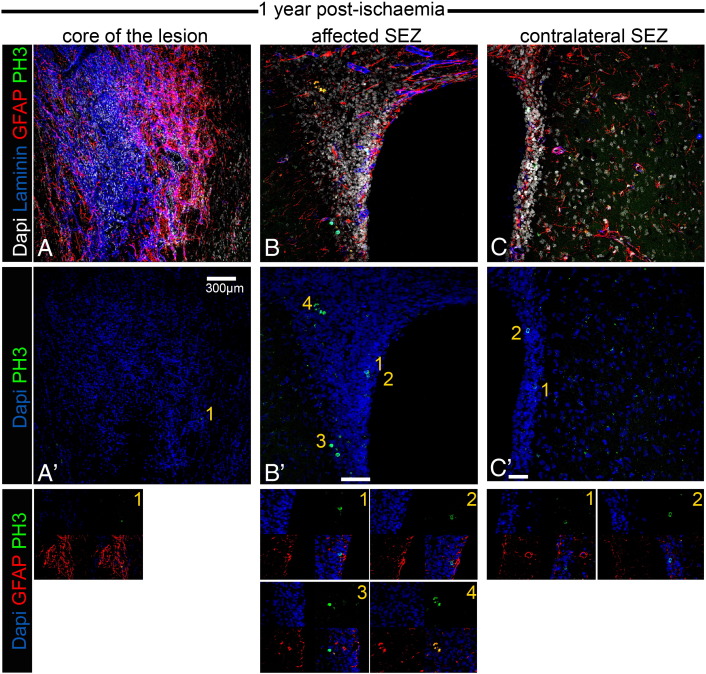
Occurrence of mitotic GFAP + cells 1 year post-ischaemia. Microphotographs showing stacks of images taken from thick coronal sections of rat brain tissue 1 year after MCAO. As in Fig. S3, the ischaemia-affected area in the striatum (A) is characterized by high levels of immunostaining for GFAP (in red, marking astroglial cells) and for laminin (in blue, marking swelled blood vessels and deposition in the parenchyma). In B, a part of the adjacent to the lesion SEZ is depicted characterized by gliosis and blood vessel swelling, as well as by a marked expansion of cell numbers, when compared to the unaffected contralateral hemisphere (in C). In the middle panel the same areas are shown only with DNA counterstaining of nuclei (in blue) and with immunostaining for PH3 (in green). Note the rarity of mitotic figures in the gliotic area of lesion, compared to increased PH3 + cells in the affected SEZ. Mitotic activity is also high (but lower that in the affected SEZ) in the contralateral SEZ. In the examples of mitotic cells shown in the lower panel we have included an immunostaining artefact (in No. 4 in B′ immunopositive spots have no nuclei). [White bars indicate the width of the SEZ].

The use of GFAP and PH3 to identify mitotic NSC introduces the possibility that, as all mitotic astrocytes will be included in this population, any increase in the proliferation of the non-NSC astrocyte population with age or injury (due to gliosis) will be mis-interpreted as an increase in NSC proliferation. To investigate this we compared the numbers of PH3 +/GFAP + cells in the ageing (1 year post-sham operation) unaffected SEZ and in the young unaffected SEZ. We found no significant differences (Supplementary Table and Figs. S3C, S4C), indicating the absence of age-related gliotic proliferation in the niche. We also counted the numbers of mitotic GFAP + cells in the highly gliotic penumbra of our lesions within the adjacent striatum, a region where no NSC would be expected. Here we found very few GFAP +/PH3 + cells — the density of these mitotic astrocytes was approximately 5 times lower than the density of PH3 +/GFAP + cells in the cytogenic niche and they constituted less than 3.5% of the total PH3 + cells (Supplementary Table and Figs. S3, S4), in agreement with previously published reports (Li et al., 2010b). We conclude, therefore, that any increase in astrocyte mitosis due to age and/or injury would make only a minimal contribution to the numbers of GFAP +/PH3 + cells in the post-stroke SEZ, and that the great majority of these cells are mitotic NSC.

Having validated the use of GFAP and PH3 labelling to identify and quantify mitotic NSC, we examined the parts of the SEZ directly affected by ischaemia 4–5 weeks and 1 year after stroke. The numbers of mitotic NSCs showed approximately an 8-fold increase at 4–5 weeks post-injury and had returned to normal levels at 1 year post-injury (Fig. 3), whilst numbers of mitotic progenitors were significantly increased at both post-ischaemic phases (Fig. 3). Notably, in the domains of the niche not affected by ischaemia the mitotic behaviour of neural stem and progenitor cells remained normal at both time-points (Figs. 1, 3).

**Fig. 4:**
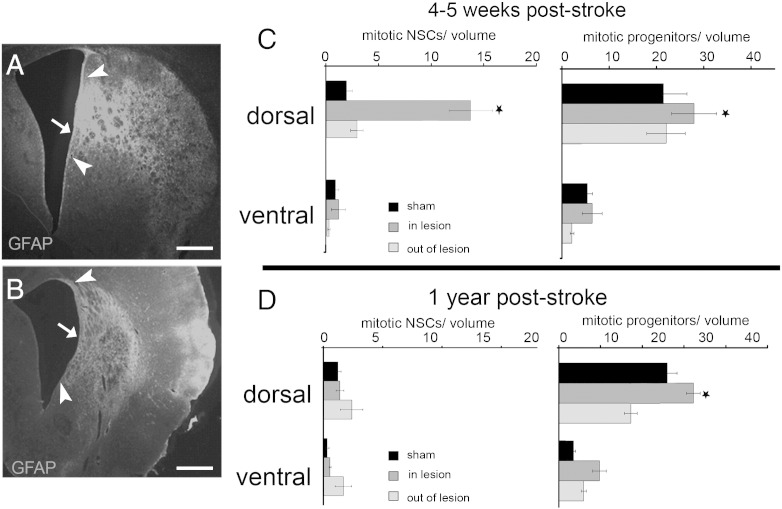
The effect of position in the response of the SEZ to ischaemia. (Panels A, B) Coronal sections of ischaemic rat brains immunostained for GFAP. These images are the ones shown in Fig. 3 with the addition of the sub-division of the SEZ in dorsal and ventral halves (indicated by the arrow; arrowheads indicate the directly affected area of the SEZ). (Panels C, D) Graphs showing the density of mitotic neural stem and progenitor cells within the two halves of the SEZ in sham-operated and ischaemic rats, 4–5 weeks (C) and 1 year (D) after the insult. Densities are shown separately for areas directly affected by ischaemia and for non-affected areas (grey and light grey bars, respectively). [volume = 1 mm (length) × 70 μm (section thickness) × 200 μm (width from the ventricular wall) = 0.014 mm^3^; * = p < 0.05, compared to the respective normal value (black bars), using two-way ANOVA. Error bars depict SEM. Scale bars: 500 μm].

The SEZ is not homogeneous in terms of density, activity and developmental origin of NSCs (Merkle et al., 2007; Mirzadeh et al., 2008; Young et al., 2012) and in the mouse we have previously documented higher levels of mitotic activity in the dorsal part of the niche (Kazanis et al., 2010); i.e. the part in which the generation of olfactory bulb neurons continues throughout life in the “homeostatic” function of the niche (Luo et al., 2006). A similar gradient of mitotic activity was observed for both progenitor pools in the homeostatic rat niche with the occurrence of mitoses significantly decreasing in the ventral half of the SEZ (Figs. 1, 4). Strikingly, although ischaemia significantly induced the mitotic activity of either the NSC or the progenitor populations of the dorsal half of the niche, it failed to activate neural stem and progenitor cells residing in the ventral half even when the area of damage extended into this ventral region (Fig. 4).

**Fig. S5:**
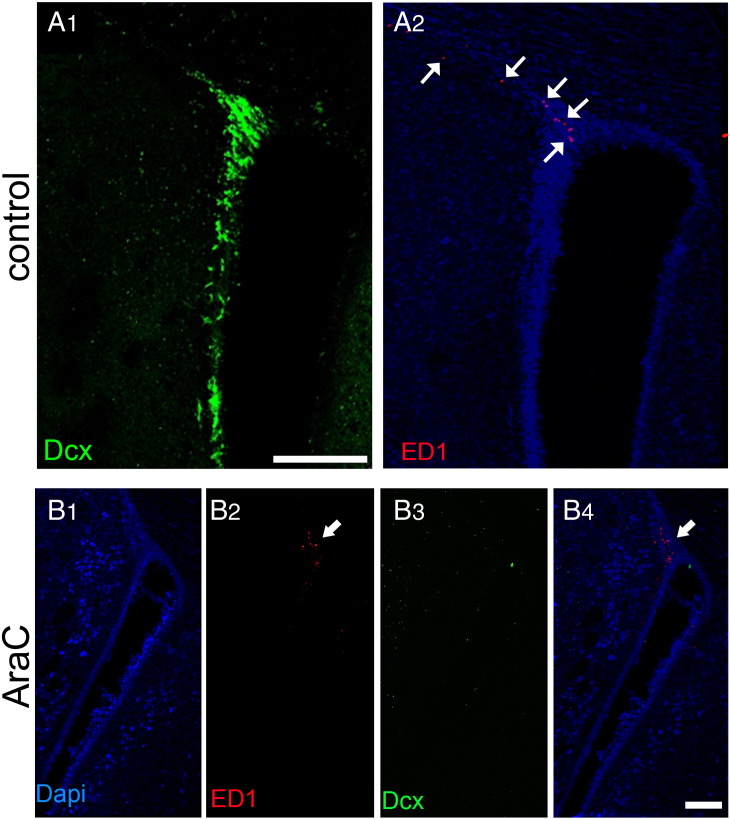
Distribution of ED-1 + cells in normal rat brain and after AraC treatment. (Panel A) Double immunostaining of a coronal section from a sham-operated rat brain showing Dcx + neuroblasts within the SEZ (A1) and the absence of ED1 + activated macrophages and microglia, with the exception of the area at the dorsal–lateral tip of the ventricle that includes the initial fragment of the rostral migratory stream (arrows in A2). (Panel B) Double immunostaining of a coronal section from an AraC-treated rat brain showing that the SEZ is depleted of Dcx + neuroblasts (B3) and the absence of ED1 + activated macrophages and microglia, again with the exception of the domain at the dorsal–lateral tip of the ventricle and the initial fragments of the rostral migratory stream (arrows in B2 and B4). [Scale bar: 200 μm in panel A; 100 μm in panel B].

In order to assess the strength of the response of NSCs after ischaemia, we compared it with an experimental condition in which the NSC pool of the niche is stimulated to become highly mitotic and that we used as an index of the maximal endogenous capacity of SEZ NSCs for mitotic activation. To achieve this, a group of rats (n = 4) received intra-cerebral infusions of the anti-mitotic drug Cytosine β-d-arabinofuranoside (AraC), via a mini-osmotic pump, for 7 days and animals were sacrificed 2 days later. Previous experimental work has shown that the infusion of AraC results in the ablation of virtually all actively dividing progenitors in the SEZ, leading to the loss of Dcx + cells shown in Fig. S5B and stimulates the mitotic activity of NSCs that quickly regenerate the niche (Doetsch et al., 1999). We have previously estimated that almost all NSCs enter the cell cycle during post-AraC regeneration in the mouse (Kazanis et al., 2007) and that the rat SEZ responds in a similar way (Kazanis and ffrench-Constant, 2011). As expected, 2 days post-AraC treatment, the majority of mitotic cells were GFAP + (n = 98 PH3 + cells analysed in total, 65 of them were GFAP +) and their numbers were significantly higher compared to control rats (approximately 6 times more, Fig. 3). Our analysis revealed that the level of mitotic activation of NSCs observed in directly affected areas of the niche during the sub-acute post-ischaemic phase was similar to the activation of NSCs (GFAP +/PH3 + cells) after AraC treatment (Fig. 3), from which we inferred that ischaemic damage of the SEZ maximally stimulated the directly affected NSC pool.

**Fig. 5:**
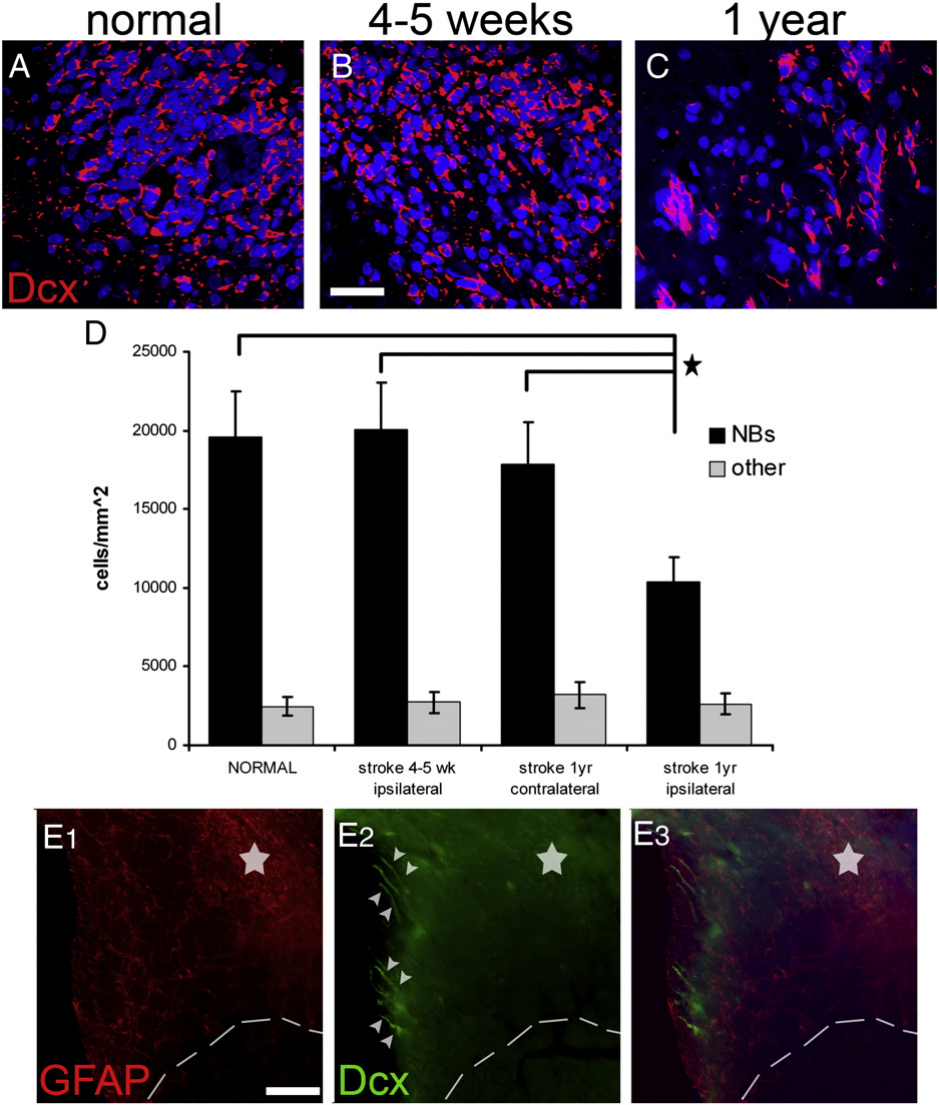
Migration of neuroblasts in the RMS after ischaemia. Coronal sections from the anterior forebrain of normal (A) and post-ischaemic (B, C) rats were immunostained for Dcx. At these levels, Dcx + cells are observed only within the RMS. The number of migrating Dcx + cells remained normal at the sub-acute phase post-injury (compare A with B), but was decreased at 1 year post-ischaemia (C). (D) Graph showing the quantification of cells in the RMS in sham-operated and post-ischaemic rats. Note that the Dcx negative fraction of cells (possibly the structural components of the stream) is not affected by ischaemia. (Panel E) Coronal section from the forebrain of a rat 1 year post-ischaemia immunostained for Dcx (in green) and GFAP (in red), showing chains of neuroblasts (indicated by white arrowheads) ectopically directed towards the affected area (delineated by the interrupted line, the star indicates the core of the lesion in the striatum). [Scale bar: in A, B 30 μm; in E 100 μm; *: p < 0.05 using one-way ANOVA, error bars represent SEM].

The normal function of dividing neurogenic NSCs in the SEZ is to generate cells (neuroblasts) that migrate along the rostral migratory stream (RMS) to the olfactory bulb where they integrate into the circuitry of that structure (Lois et al., 1996). In order to assess if this process is affected by ischaemia, coronal vibratome-cut sections were collected from the rostral part of the forebrain (that contains the RMS) and the number of migrating neuroblasts (Dcx + cells) was counted. No difference in the number of Dcx + cells within the RMS was observed at 4–5 weeks post-ischaemia; however, their number was significantly decreased (almost to half) at 1 year post-surgery (Fig. 5). Interestingly, the number of Dcx-negative cells of the RMS was normal at this time-point, suggesting that the structural components of the RMS (mainly astrocytes) were not affected either directly by injury or as a consequence of the decreased flow of neuroblasts, or ageing (Fig. 5).

#### Ischaemia-induced alterations in the cyto-architecture of the neurogenic niche

Our analysis revealed a tight spatial regulation of the response of the neurogenic niche to ischaemia, such that enhanced mitotic activation of neural stem and progenitor cells was observed only within the directly affected domains of the SEZ. Previous studies in rodents have shown that in the SEZ the positioning of progenitors next to the ventricular wall and around blood vessels is not random and that it might impact their proliferation and migration behaviour (Kazanis and ffrench-Constant, 2011; Kazanis et al., 2010; Mirzadeh et al., 2008; Shen et al., 2008; Tavazoie et al., 2008). We therefore sought to investigate how different was the cyto-architecture of the niche in the affected domains in which mitotic activity was significantly increased post-ischaemically, as compared to their neighbouring unaffected ones. In young control adult rats (analysed 4–5 weeks post-sham surgery), we observed a close spatial relationship between mitotic progenitors and both ependymal cells and blood vessels, with 38% of mitoses observed at distances smaller than 15 μm from the ventricular wall (i.e. adjacent to the ependymal cell layer) and 28.2% located within a distance of 10 μm from the nearest blood vessel (n = 86 cells analysed, average distances are shown in Table 1). In post-ischaemic animals analysed at the respective time-point we found no difference in the distribution of mitoses around the vasculature and the ventricular wall both within and outside the directly affected areas (n = 321 cells analysed, Table 1). Interestingly though, ischaemia was found to impact on the cyto-architecture of the ageing post-ischaemic niche. In the sham operated group analysed one year after the insult (i.e. in older rats), mitotic activity occurred significantly farther away from the ventricle than in the normal younger animals (those analysed 4–5 weeks after sham operation), with only 8% of progenitors found to be dividing adjacent to the ependymal cells (n = 53 cells analysed, Table 1). However, in lesioned animals and within areas of the SEZ directly affected by ischaemia the enhanced mitotic activity of progenitors described above occurred nearer to the ventricle with the percentage of progenitors dividing near ependymal cells being similar to young rats (n = 178 cells analysed, 34% of mitotic progenitors were located up to 15 μm from the ventricle). These data reveal that stroke induced an alteration in the anatomy of neurogenesis in ageing rats having survived 1 year after the insult, with the architecture of mitoses in the domains of the niche showing signs of sustained activation resembling that of a younger niche. In contrast, the distribution of mitotic progenitors around blood vessels was not affected by ageing and also remained unaltered by ischaemia (Table 1).

**Fig. 6:**
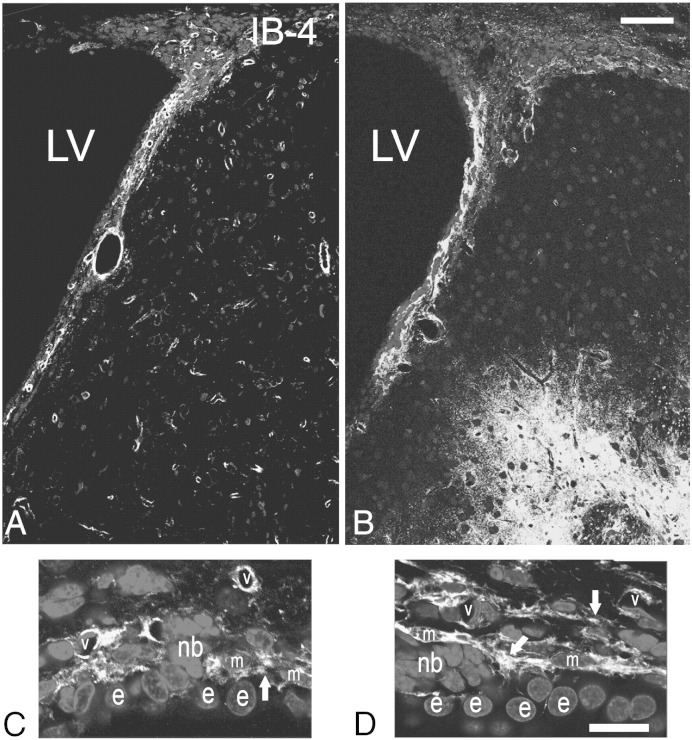
Expression of IB-4 in the normal and post-ischaemic rat brain. Coronal sections from a sham-operated (A), and an ischaemic rat at 5 weeks post-injury (B) were stained for isolectin B-4 (IB-4). Expression of IB-4 was increased within the post-ischaemic SEZ, whilst high numbers of IB-4 + cells appeared ectopically within the damaged striatal tissue (lower right part of the image). (Panels C and D) High magnifications of the SEZ from a sham-operated (C) and a post-ischaemic (D) rat, reveal that ischaemia resulted in increased expression of IB-4 due to the appearance of more IB-4 + processes (white arrows). [LV: lateral ventricle; e: ependymal cell; m: macrophage; nb: cluster of neuroblasts; v: blood vessel; scale bars: 200 μm in A, B and 15 μm in C, D].

**Fig. 7:**
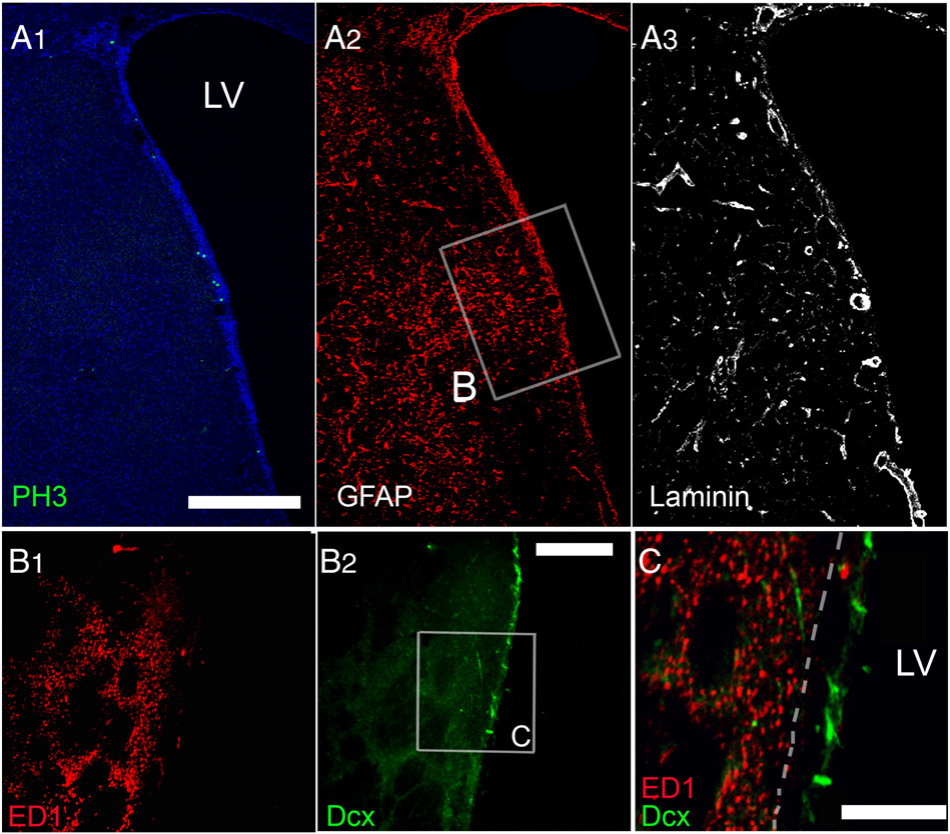
The presence of phagocytotic cells after ischaemia. (Panel A) Triple immunostaining of a coronal section from a rat brain 4 weeks post-ischaemia. Mitotic cells (PH3 +, in green in A1) were increased in the domain of the SEZ directly affected by ischaemia, as outlined by increased expression of GFAP (A2, boxed area) and swelling of blood vessels (A3). (Panel B) High magnification of a section adjacent to that shown in panel A, focusing on the part directly affected by ischaemia (respective to the area boxed in A2). Immunostaining for ED1 and Dcx shows that macrophages are not observed within the SEZ, which is defined by the presence of Dcx + cells. A detail of the area boxed in B2 is presented in C with the limits of the SEZ shown with a white interrupted line. [Scale bar: 450 μm in panel A; 200 μm in panel B; 100 μm in C].

Another population of cells present in the SEZ and postulated to play a role in the neurogenic response to tissue-damage are cells of the immune system, either microglia (Walton et al., 2006) or blood-derived macrophages (Thored et al., 2009). In order to investigate how differently do these cells behave within and out of the directly affected domains of the niche, sections were stained for isolectin B-4 (IB-4), that labels all microglia and macrophages, or were immunostained for ED1, a marker of activated phagocytotic cells of the immune system. In sham-operated rats (i.e. in the homeostatic niche) the distribution of IB-4 labelled-cells in the SEZ followed the dorso-ventral gradient of mitotic cells, with higher densities observed dorsally (Fig. 6A). Consistent with previous reports that IB-4 labels endothelial cells (Grossmann et al., 2002), a fraction of IB-4 immunoreactivity was detected perivascularly within the SEZ, expressed either by cells surrounding blood vessels or by blood vessel mural cells (Figs. 6C, D and S7) and another fraction by cells within the parenchyma, whilst outside the niche only perivascular IB-4 immunoreactivity could be observed (Fig. 6A). Interestingly, in the ageing SEZ the density of IB-4 + cells was significantly decreased (Table 1). ED1 + cells were not observed in the homeostatic young or aged niche with the exception of the domain adjacent to the dorsal–lateral tip of the ventricle and the initial fragment of the RMS (Figs. S5A and S7A). 4–5 weeks and 1 year after ischaemia, expression of IB-4 was increased, compared to the respective controls, throughout the SEZ as a result of the emergence of more IB-4 + processes, but cell numbers were not changed (Table 1 and Fig. 6). Outside the SEZ numbers of IB-4 + cells were markedly increased specifically in the damaged striatal and cortical tissue (Figs. 6 and S1). ED1 + phagocytotic cells were widespread throughout the entire lesion outside the SEZ at 4–5 weeks and 1 year post-surgery; however, only sparse ED1 + cells were observed within the affected or unaffected SEZ (Figs. 7 and S7), again with the exception of the dorsal-most part and the RMS where their numbers remained at normal levels (data not shown). In order to compare with the regenerating SEZ, when NSCs are maximally activated, sections taken from rat brains 2 days after the end of AraC treatment were also stained for IB-4 and ED1. The number of IB-4 + cells, the expression levels of IB-4 and the number and distribution of ED1 + cells all remained identical to those of the homestatic niche (Fig. S5 and data not shown).

## Discussion

Experimental animal studies as well as post-mortem examinations of human patients have indicated that the SEZ neurogenic niche responds to brain tissue damage such as striatal ischaemic injury. This response includes both activation — an increase of proliferation within the niche (Gao et al., 2008; Jin et al., 2001, 2006; Komitova et al., 2005; Li et al., 2010a; Marti-Fabregas et al., 2010; Sgubin et al., 2007; Thored et al., 2006; Zhang et al., 2004) and the directed migration of newborn cells (neuronal and glial progenitors) towards the areas of damage (Ekonomou et al., 2010; Hou et al., 2008; Thored et al., 2006, 2007; Yamashita et al., 2006). Although the degree of subsequent neuronal integration and tissue repair is very poor, recovery is even further compromised in the absence of this activation and endogenous neurogenesis (Jin et al., 2010; Sun et al., 2012; Wang et al., 2012). Understanding the signals that initiate and sustain activation in the SEZ is therefore important, not least because enhancing the endogenous response might further improve any functional recovery. Here we have focused the spatio-temporal and cellular characteristics of activation in the sub-acute and chronic phases after a focal ischaemic insult, stages that have been poorly investigated thus far but which would represent important clinical targets for regenerative medicines in stroke (Markus et al., 2005).

We have made three key observations. First, mitotic activation of neural stem and progenitor cells occurs only within the areas of the niche directly affected by ischaemia. A similar correlation between the activation of the SEZ and the proximity of the lesion has been described before after cortical injury (Goings et al., 2006) and in experimental and human cases of stroke (Ekonomou et al., 2010; Shimada et al., 2010). Second, activation is only seen in the dorsal SEZ. This suggests that cell-cycle regulation is also dependent on intrinsic mechanisms and highlights the mosaic nature of NSCs (Merkle et al., 2007; Mirzadeh et al., 2008; Young et al., 2012). We hypothesise that these intrinsic mechanisms promote senescence of ventral cells. Previous work has revealed that the age-dependent shrinkage of the SEZ is initiated at the ventral domains and extends dorsally (Luo et al., 2006) and we have observed increased histochemical staining for senescence-associated β-gal activity (SA-β-gal), a specific marker of cellular senescence (Campisi and d'Adda di Fagagna, 2007) in the ventral domain even in young adult rats (our unpublished data). Further investigations of the generation of calbindin + olfactory bulb neurons (that are preferentially generated from ventral SEZ progenitors) (Merkle et al., 2007) would test this hypothesis. As a result of this limitation of activation to dorsal regions, the fraction of the SEZ that responded to damage varied from 15% (observed in a rat at 5 weeks post-insult) (Fig. 2A), to 74% (in a rat at 1 year post-insult) (Fig. 2B). Third, the responding cell populations were different in the two time-points studied, with NSCs and progenitors both being activated in the sub-acute phase but only progenitors still being mitotically active at the chronic phase. However NSC activation at the earlier time-point was maximal, as shown by comparing the level of activation after ischaemia with that seen when they efficiently and rapidly regenerate the amplifying precursor population niche after ablation of the latter population with AraC. Recent experimental work in other adult stem cell systems (such as the intestine and the epidermis) has highlighted the importance of the distinct activation of stem and progenitor cells in tissue maintenance and regeneration (Mascre et al., 2012; Simons and Clevers, 2011).

In addition to any intrinsic mechanisms responsible for senescence in the ventral SEZ, our data point to two sets of extrinsic signals that regulate SEZ behaviour post-stroke. First, those that reverse the changes in the location of mitoses associated with ageing. When we mapped the position of mitotic neural stem and progenitor cells of the SEZ with respect to the major structural and regulatory components of the niche – ependymal cells, blood vessels and microglia – we found that mitoses occur at close proximity to the ependymal cell layer/cerebrospinal fluid interface and to blood vessels in the normal and the activated SEZ, alike, consistent with previous conclusions that signals derived from ependymal and endothelial cells promote mitoses (Colak et al., 2008; Gajera et al., 2010; Mirzadeh et al., 2008; Shen et al., 2008; Tavazoie et al., 2008; Walton et al., 2006). However we also found that the distance of mitotic cells from the ventricular wall increased with ageing, in agreement with recent reports (Shook et al., 2012) and that these changes are reversed in the injured dorsal SEZ to restore the architecture towards that seen in the younger niche, possibly reflecting a weakening with ageing and enhancement following injury of putative pro-mitotic signals derived from cerebrospinal fluid (Johanson et al., 2008) and/or ependymal cells (Luo et al., 2006, 2008). Second, the spatial restriction of the increase in mitoses within the ischaemia-affected areas points to a set of proliferative signals, derived from the lesion and acting over short distances, also controlling activation of NSCs. Multiple candidate molecules have been identified as possible mediators of stroke-derived activation of neurogenesis, progenitor migration and differentiation (Madri, 2009; Robin et al., 2006; Thored et al., 2009; Wang et al., 2011; Yan et al., 2007) and non-diffusible factors, such as the extracellular matrix, might play a role in limiting the spatial response of the stem and precursor cells to any diffusible signals.

We also examined the non-NSC-lineage cells of the niche to determine the potential contribution of macrophages/microglia to SEZ activation following ischaemia. Previous experimental work has showed that the innate and blood-born immune cell system is activated after brain injury (Thored et al., 2009), although depending on the position of the lesion (Goings et al., 2006), after ischaemia it still remains unclear whether this activation exerts a pro-neurogenic (Walton et al., 2006), an anti-neurogenic (Hoehn et al., 2005), or a neutral (Thored et al., 2009) role. In our analysis, we found that focal ischaemia induced the expression of IB-4 within the affected parts of the niche, in concert with previous reports (Thored et al., 2009), although as a result of sprouting of new processes rather than increased numbers of cells. Phagocytosis, as judged by expression of ED1, was not observed within the normal, the post-ischaemic, or the regenerating niche, with the exception of the initial fragments of the RMS, i.e. areas of intense neuroblast migration. The limited activation of cells of the immune system within the SEZ in contrast to the marked presence of phagocytotic cells within the normal migratory route of the RMS and at sites of lesion in the striatum possibly reflects the absence of marked structural reorganization within the niche, as revealed by the lack of changes in the distribution of mitoses in respect to blood vessels, and is in agreement with the previously suggested hypothesis that the major role of SEZ microglia/macrophages is to provide local trophic support, in contrast to cells of the immune system at sites of lesion that are necessary both for support and for tissue remodelling (Thored et al., 2009).

Taken together, our data have implications for therapeutic strategies designed to enhance neurogenesis in stroke. We have identified significant limitations to the endogenous regenerative response of the SEZ at post-acute time-points post-injury; dependency on direct involvement in the lesion and a loss of NSC activation at later time-points. It follows that strategies to enhance regeneration by addition of growth factors, already known to enhance the ischaemia-induced increase of SEZ neurogenesis (Chen et al., 2005; Liu et al., 2007, 2009; Thored et al., 2006), might be further improved by targeting either uninvolved NSC or stem cell quiescence. An additional point highlighting the need to enhance endogenous neurogenesis after a stroke is the observed significant reduction of neuroblast migration to the olfactory bulbs one year post-insult. Whilst the generation of olfactory neurons throughout life may be of less significance in humans than in rodents and other macrosmatic species, the long-term perturbation of the SEZ neurogenic output post-ischaemia, possibly due to ectopic migration of progenitors (Fig. 5E), revealed by our results might lead to additional side-effects even in humans.

## Sources of funding

This work was funded by an NIH—National Institute of Biomedical Imaging and Bioengineering Quantum Grant Project [1P20EB00706] to ΜΜ and Cff-C, a BBSRC UK grant (BB/I013210/1) to RF and IK and by a Wellcome Trust, Value in People fellowship to IK.

## Disclosures

There are no conflicts of interest to be disclosed.

The following are the supplementary data related to this article.

Supplementary Table Table showing the density of mitotic GFAP + cells as well as their contribution to the total pool of PH3 + cells, in the affected and the contralateral (unaffected) SEZ and in the adjacent penumbra of the lesion (in the striatum). Note that the density of mitotic GFAP + cells in the niche is significantly increased only at the 4–5 weeks post-ischaemia time-point. Also, note that the occurrence of PH3 +/GFAP + cells (considered to be dividing gliotic astrocytes) in the penumbra is minimal in both time-points, even when compared to the unaffected SEZ. Within the penumbra the vast majority of mitotic cells (more than 95%) is consisted of microglia and macrophages. [**: p < 0.01 comparing the density of PH3 +/GFAP + cells in the affected SEZ at 4–5 weeks post-ischaemia with all other time-points and areas. *: p < 0.05 comparing the density of PH3 +/GFAP + cells in the penumbra of the lesion at 4–5 weeks and at 1 year post-ischaemia with the other time-points and areas. Statistical analysis was performed using one-way ANOVA followed by the Bonferroni post-hoc test].

**Fig. S6:**
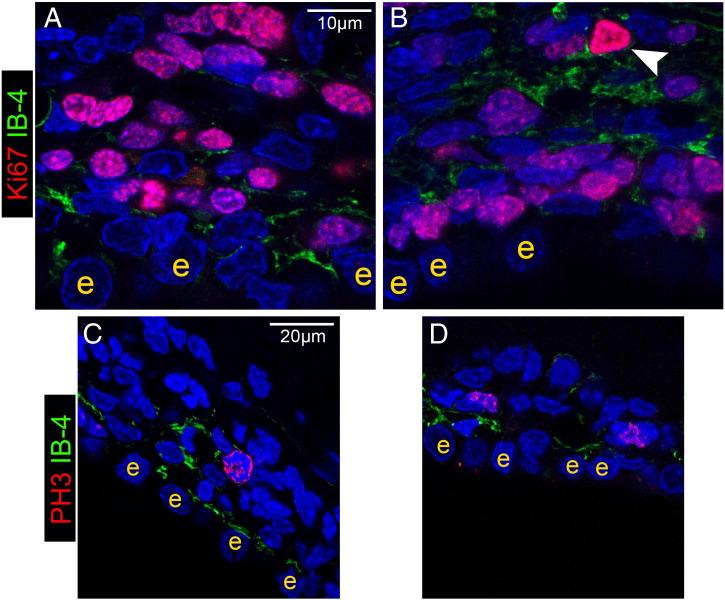
Proliferating microglia/macrophages in the post-ischaemic SEZ. Figure showing immunostained areas of the SEZ in high magnification. The vast majority of proliferating cells (marked with Ki67 + in A and B, or PH3 in C and D, all in red) is negative for IB-4 (in green in all photos), either in sham-operated (A and C), or in post-ischaemic (4 weeks after the insult, B and D) rats. One exception is highlighted by the white arrowhead in B. [e: ependymal cell].

**Fig. S7:**
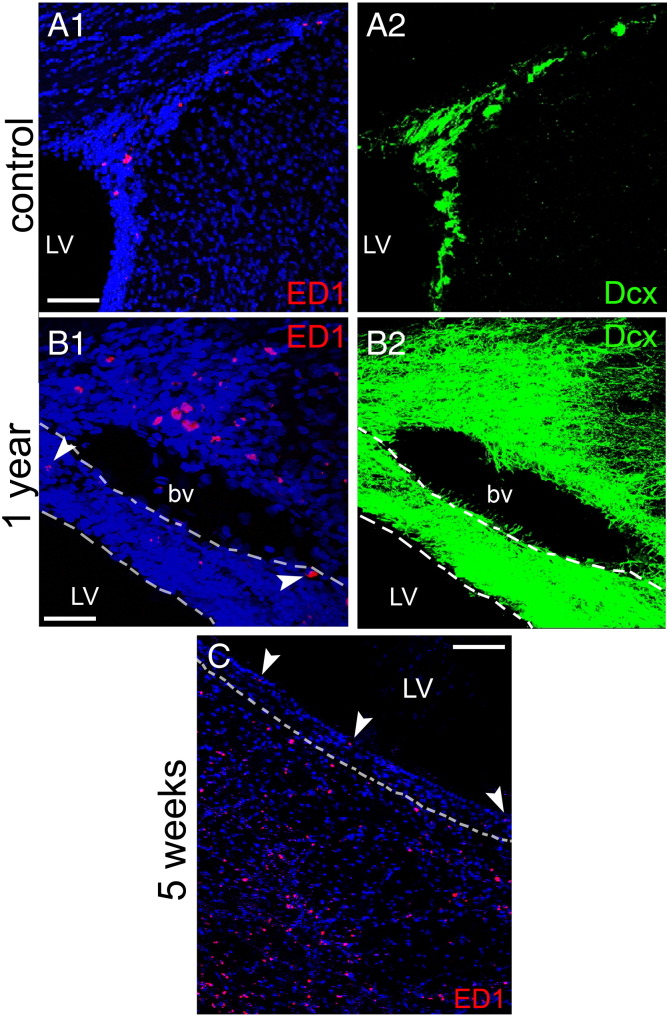
Distribution of ED-1 + cells after ischaemia. (Panels A, B) Double immunostainings of coronal sections from a sham-operated rat brain (panel A) and a post-ischaemic rat (1 year after the insult, in panel B), for Dcx + neuroblasts (in green) and ED1 + phagocytotic cells of the immune system (in red). Note that ED1 + activated macrophages and microglia are virtually absent from the SEZ (A1, B1) with the exception of the area at the dorsal–lateral tip of the ventricle that includes the initial fragment of the rostral migratory stream and few cells indicated by arrowheads in B1. Also note the highly activated state of the SEZ (highlighted by the white interrupted lines) in panel B, as evident by the significant increase in the presence of Dcx + immature neurons. Nevertheless, ED1 + cells are largely observed only outside the niche, at the distant wall of the blood vessel, adjacent to the area of lesion. (Panel C) Immunostaining of a coronal section from a post-ischaemic rat (5 weeks after the insult) for ED1 + (in red), again showing the dense presence of ED1 + cells within the affected striatum, in contrast to few positive cells within the adjacent SEZ (highlighted by the white interrupted line). [Scale bars, 200 μm in A and C; 100 μm in B; LV: lateral ventricle, bv: blood vessel].

## Figures and Tables

**Table 1:** This table shows different parameters of the cyto-architecture of the homeostatic (measured in sham-operated rats) and the post-ischaemic SEZ. Columns 4 and 5 show the average shortest distance of all mitotic nuclei from the ventricular wall and the nearest blood vessel, respectively. Column 6 shows the number of IB-4 + cells expressed per length of ventricular wall. Note the significant increase in the average distance of mitotic nuclei from the ventricle in the ageing SEZ (15 month old homeostatic niche) and the reversal of this phenotype within the ischaemia-affected domains (15 month old, 1 year post-lesion niche). Also note the decrease in the density of IB-4 + cells in the ageing SEZ (15 month old homeostatic niche). Finally, note the absence of changes in the perivascular distribution of mitotic nuclei (column 5).

Condition	Age	Domain	Distance (μm): ventricle	Distance (μm): blood vessel	IB-4 + cells/length
Homeostasis	4 months (4–5 weeks post-sham operation)	Dorsal	30.9 ± 24.6	14.8 ± 10.2	65.98 ± 12.35
Ventral	28.6 ± 21.4	16.3 ± 11.5	50.92 ± 15.23
15 months (1 year post-sham operation)	Dorsal	53.1 ± 25.7⁎⁎⁎	18.5 ± 10.9	35.60 ± 8.78⁎⁎⁎
Ventral	49.3 ± 20.6⁎⁎⁎	17.3 ± 9.1	28.23 ± 9.64⁎⁎⁎
Post-ischaemic	4 months (4–5 weeks post-lesion)	Dorsal in the lesion	22.5 ± 20.6	17.1 ± 9.6	38.27 ± 21.37
Dorsal outside	25.4 ± 22.7	16.6 ± 9.6	46.54 ± 17.77
Ventral in the lesion	27.0 ± 20.1	15.3 ± 8.9	54.29 ± 12.90
Ventral outside	23.6 ± 18.6	18.2 ± 10.1	31.65 ± 19.22
15 months (1 year post-lesion)	Dorsal in the lesion	29.9 ± 22.6†††	22.4 ± 14.1	40.43 ± 13.45
Dorsal outside	51.3 ± 27.1	18.2 ± 11.1	23.61 ± 13.54
Ventral in the lesion	32.8 ± 21.1†††	17.9 ± 10.0	34.21 ± 10.65
Ventral outside	47.9 ± 20.6	19.4 ± 9.9	30.77 ± 9.32

⁎⁎⁎ p < 0.05 using two-way ANOVA and comparing aged and young homeostatic rats.

††† p < 0.05 using two-way ANOVA and comparing aged homeostatic rats and post-ischaemic rats 1 year after the insult.
