# Supplementary material for: The late response of rat subependymal zone stem and progenitor cells to stroke is restricted to directly affected areas of their niche
Source: Exp Neurol. 2013 Oct;248:387–97. doi: 10.1016/j.expneurol.2013.06.025 (PMC3782662; doi:10.1016/j.expneurol.2013.06.025)
Supplement: Supplementary Table — Table showing the density of mitotic GFAP + cells as well as their contribution to the total pool of PH3 + cells, in the affected and the contralateral (unaffected) SEZ and in the adjacent penumbra of the lesion (in the striatum). Note that the density of mitotic GFAP + cells in the niche is significantly increased only at the 4–5 weeks post-ischaemia time-point. Also, note that the occurrence of PH3 +/GFAP + cells (considered to be dividing gliotic astrocytes) in the penumbra is minimal in both time-points, even when compared to the unaffected SEZ. Within the penumbra the vast majority of mitotic cells (more than 95%) is consisted of microglia and macrophages. [**: p < 0.01 comparing the density of PH3 +/GFAP + cells in the affected SEZ at 4–5 weeks post-ischaemia with all other time-points and areas. *: p < 0.05 comparing the density of PH3 +/GFAP + cells in the penumbra of the lesion at 4–5 weeks and at 1 year post-ischaemia with the other time-points and areas. Statistical analysis was performed using one-way ANOVA followed by the Bonferroni post-hoc test]. [file mmc1.doc]

**Supplementary Table**

|  | Number of PH3+/GFAP+ cells per volume | Percentage of GFAP+/PH3+ cells per total PH3+ cells |
| --- | --- | --- |
| Affected SEZ |  |  |
| 4-5 weeks | 4.78±1.23 ****** | 21.46% |
| 1 year | 1.02±0.35 | 6.58% |
| Contralateral SEZ |  |  |
| 4-5 weeks | 1.36±0.74 | 7.19% |
| 1 year | 0.58±0.29 | 8.14% |
| Penumbra of the lesion |  |  |
| 4-5 weeks | 0.21±0.15 ***** | 3.25% |
| 1 year | 0.12±0.09 ***** | 2.09% |

Table showing the density of mitotic GFAP+ cells as well as their contribution to the total pool of PH3+ cells, in the affected and the contralateral (unaffected) SEZ and in the adjacent penumbra of the lesion (in the striatum). Note that the density of mitotic GFAP+ cells in the niche is significantly increased only at the 4-5 weeks post-ischaemia time-point. Also, note that the occurrence of PH3+/ GFAP+ cells (considered to be dividing gliotic astrocytes) in the penumbra is minimal in both time-points, even when compared to the unaffected SEZ. Within the penumbra the vast majority of mitotic cells (more than 95%) is consisted of microglia and macrophages. [**: p<0.01 comparing the density of PH3+/GFAP+ cells in the affected SEZ at 4-5 weeks post-ischaemia with all other time-points and areas. *: p<0.05 comparing the density of PH3+/GFAP+ cells in the penumbra of the lesion at 4-5 weeks and at 1 year post-ischaemia with the other time-points and areas. Statistical analysis was performed using one-way ANOVA followed by the Bonferroni post-hoc test]
